# Apolipoprotein E (ApoE) orchestrates adipose tissue inflammation and metabolic disorders through NLRP3 inflammasome

**DOI:** 10.1186/s43556-023-00158-8

**Published:** 2023-12-08

**Authors:** Yulin Zhang, Ziwei Cheng, Liyu Hong, Jia Liu, Xinyue Ma, Wenjing Wang, Ran Pan, Wenjie Lu, Qichao Luo, Shan Gao, Qin Kong

**Affiliations:** 1 Department of Pharmacology, School of Basic Medical Sciences, Anhui Medical University, Hefei, 230032 China; 2 The Second Clinical Medical College, Anhui Medical University, Hefei, 230032 China; 3 RainbowFish Rehabilitation and Nursing School, Hangzhou Vocational and Technical College, Hangzhou, 310000 China

**Keywords:** Apolipoprotein E (ApoE), Adipose tissue, Metabolic inflammation, Macrophage, NOD-, LRR- and Pyrin Domain-Containing Protein 3 (NLRP3)

## Abstract

Obesity is a metabolic disorder characterized by the hypertrophy expansion of adipose tissue, resulting in dysregulated energy metabolism, and accompanied by chronic low-grade inflammation. Adipose tissue macrophages (ATMs), a principal component of inflammation, respond to microenvironment signals and modulate adipose tissue remodeling and metabolic processes situation-specific. However, the mechanisms governing how the organism maintains equilibrium between its chronic inflammation and metabolism still need to be understood. Here, we describe a novel role of apolipoprotein E (ApoE), which associated with lipid particles, in maintaining fat deposition and system metabolic inflammation. Using human samples and mouse models, we show that ApoE is robustly downregulated in obese individuals, *db/db* mice, and mice of high-fat diet (HFD) feeding and increased in obese subjects with diabetes. Furthermore, we found that ApoE deficiency mice globally prevented obesity by restraining adipose tissue expansion and improved systemic glucose tolerance and insulin sensitivity. However, macrophage contributed to metabolic inflammation due to increased IL-1β production in adipose tissue from ApoE^-/-^ mice induced by HFD. Our results suggest that the role of ApoE in regulating obesity and obesity-associated glucose dysregulation is inconsistent. Mechanistically, ApoE modulates of the NOD-, LRR- and pyrin domain-containing protein 3 (NLRP3) inflammasome priming and activation step. Thus, our studies might provide new sights into ApoE, which is required for obesity-induced hyperglycemia, hyperinsulinism, and adaptive inflammation responses but diminishes the tolerance towards a subsequent metabolic inflammatory challenge. Our study shed new light on the integral role of apolipoprotein APOE in immunometabolism and adipose tissue homeostasis.

**Supplementary Information:**

The online version contains supplementary material available at 10.1186/s43556-023-00158-8.

## Introduction

Obesity triggers a persistent state of inflammation marked by immune cell infiltration and activation in metabolic organs such as adipose tissue, which can contribute to the onset of type 2 diabetes (T2D), fatty liver disease, and cardiovascular disease. Adipose tissue is a remarkably adaptable organ, and a crucial characteristic of adipose tissue is its exceptional plasticity. Although traditionally considered a reservoir for energy, we now understand that adipose tissue functions as an endocrine organ in regulating energy homeostasis, such as modulating insulin sensitivity and influencing immune responses [1, 2]. The interactions between immune cells and adipocytes are essential for adipose tissue function. Among these cells, ATMs play a critical role in orchestrating metabolic inflammation as initiators of the process by sensing metabolic cues [3]. Upon stress by environmental and metabolic stimuli, including mechanical stress, hyperlipidemia, and hyperglycemia, ATMs undergo activation and assume a pro-inflammatory state, forming distinctive crown-like structures (CLS) around dying adipocytes. Evidence suggests that CLS in white adipose tissue is inversely associated with human insulin sensitivity [4–6]. In obesity, macrophages accumulate within adipose tissue through resident proliferation and the recruitment of monocyte-derived precursors via the C-C motif chemokine receptor 2 (CCR2) pathway. Beyond obesity, the composition of immune cell populations in adipose tissue can be influenced by changes in the systemic metabolic state. For instance, fasting and exposure to cold can impact the abundance of ATMs in white adipose tissue (WAT) depots [7]. These alterations are believed to be linked to increased lipolysis and fatty acid oxidation processes. However, our understanding of the underlying mechanisms governing macrophage response to these systemic metabolic states is currently limited. Therefore, we sought to test the consistency of the underlying mechanism during chronic overnutrition, the dysregulation of glucose and lipid associated with metabolic inflammation aggravated related to macrophage.

The inflammasome is a cytosolic complex consisting of leucine-rich repeat-containing proteins (NLRs), nucleotide-binding domain or AIM2, adaptor protein ASC, and caspase-1. Activation of the inflammasome leads to caspase-1-mediated maturation and release of various pro-inflammatory cytokines, including interleukin-1β (IL-1β) and IL-18 [8]. Excessive activation of the NLRP3 inflammasome is implicated in developing several inflammatory disorders, such as T2D, atherosclerosis, and Alzheimer’s disease, underscoring the importance of its tight regulation [8–10]. While the NLRP3 inflammasome has been extensively studied recently, the cues underlying chronic inflammation and adipose tissue physiology remain obscure.

Apolipoprotein E (ApoE) is a crucial component of lipoproteins encoded by *ApoE*. Physiologically, it facilitates the binding of lipoproteins to receptors, contributing to the hepatic clearance of triglyceride-rich lipoprotein. In the absence of ApoE, mice exhibit severe hyperlipidemia, develop spontaneous atherosclerotic lesions, and have become a widely utilized animal model for studying atherosclerosis. Emerging evidence suggests that ApoE also participates in energy metabolism, potentially as a molecular link connecting adipose tissue and insulin resistance (IR) [11, 12]. Recent studies have revealed that in ApoE^-/-^ mice, hyperuricemia exacerbates aortic plaque load and cell apoptosis, which is associated with activating the NLRP3 inflammasome and caspase-1 pathway [13, 14]. Moreover, following lipid-lowering treatment, NLRP3 and IL-1β in aortic tissue from ApoE^-/-^ mice are downregulated, as well as a significant reduction in oxidative stress and cellular apoptosis [15]. These observations highlight the role of NLRP3 inflammasome as a regulator in atherosclerosis-associated cardiovascular disease. However, it remains unclear how ApoE is involved in IR and adipose chronic metabolic inflammation.

Here, we analyzed the expression of ApoE in the human adipose tissue transcriptomic database and adipose tissue of the obese mice model. We found that ApoE expression correlated negatively with body mass index (BMI). ApoE deficiency improved hyperinsulinemia and glucose tolerance in HFD feeding couples, which promoted overnutrition-induced metabolic inflammation in adipose tissue through NLRP3 inflammasome. Our studies, therefore, uncover the functional duality of ApoE balance inflammation and energy homeostasis.

## Results

### ApoE expression is decreased in obesity and inflammation

**Fig. 1:**
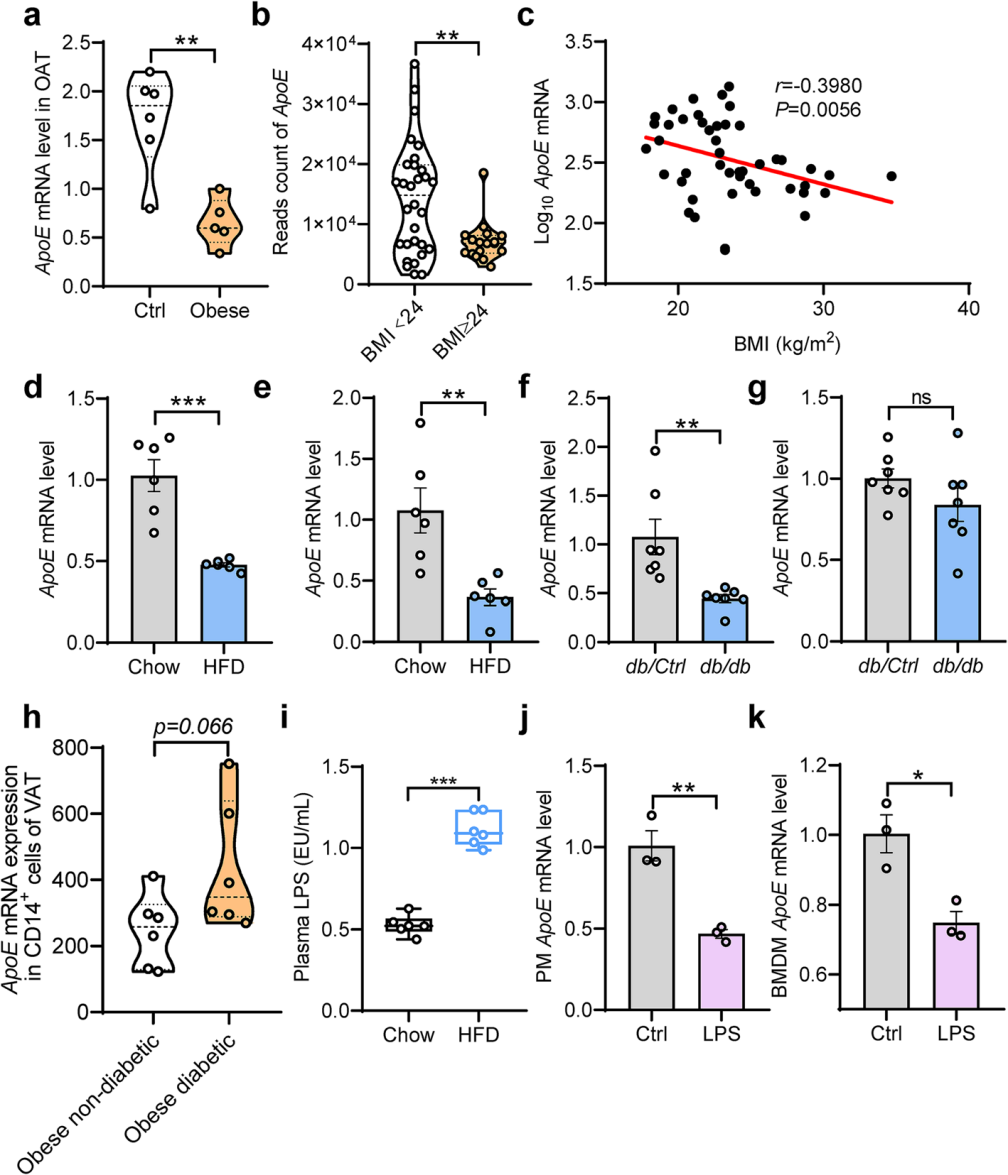
Downregulation of APOE expression in adipose tissue during obesity and inflammation. **a** Previous human samples data was collected from the GEO dataset (GSE9624, *n* = 5–6). **b** The reads count of *ApoE* in indicated groups from RNA-Seq of human OAT (*n* = 30, BMI < 24; *n* = 17, BMI ≥ 24). **c** Individual correlation between *ApoE* mRNA level and BMI value. **d**-**g** QPCR analysis of *ApoE* gene expression in eWAT (**d**, **f**) and iWAT (**e**, **g**) from WT with HFD feeding (*n* = 6) and *db/db* mice (*n* = 7). eWAT, epididymal WAT. iWAT, inguinal WAT. HFD, high-fat diet. **h** Previous human samples data of *ApoE* expression were collected from the GEO dataset (GSE54350, *n* = 6). **i** Plasma LPS levels in mice after 10-week HFD feeding (*n* = 6). **j**, **k** QPCR analysis of *ApoE* gene expression in LPS-stimulated PM (**i**) and BMDM (**j**) (100 ng/mL, 4 h, *n* = 3). VAT, visceral adipose tissue. OAT, omental adipose tissue. PM, peritoneal macrophages. BMDM, bone marrow-derived macrophages. All data represent mean ± SEM. *P < 0.05, **P < 0.01, ***P < 0.001. Significance is performed with unpaired 2-tailed Student’s *t*-test (**a**, **b**, **d**-**k**) and *Spearman’s* correlation (**c**)

To explore and understand the correlation of *ApoE* with obesity progression, we first analyzed a database of human adipose tissues from obesity (GEO accession number GSE9624) [16]. Notably, the results showed that *ApoE* was dramatically downregulated in omental adipose tissue (OAT) from obese subjects compared with the control (Fig. 1a). Next, further to evaluate the clinical relevance of *ApoE* expression with obesity, OAT samples were collected from 30 overweight/obese (BMI ≥ 24) subjects and 17 lean (BMI < 24) undergoing RNA-Sequencing (RNA-Seq) analysis (Cutoff: *P*-value < 0.05 and |Log2 (fold change) | > 0.3). As expected, the expression of *ApoE* was also significantly reduced by over 40% in obesity of human OAT (Fig. 1b). Furthermore, *ApoE* mRNA level was negatively correlated with BMI value (*r* = −0.398, *P* = 0.0056) (Fig. 1c), indicating that *ApoE* inversely associated with obesity progression. Like in the observations in human subjects, QPCR also revealed the decrease of *ApoE* gene expression in adipose tissue from the HFD-induced obesity model and genetically obese (*db/db*) mice (Fig. 1d-g). Previous studies have demonstrated that LPS plays vital role in obesity-associated chronic metabolic diseases [17, 18]. Due to the crucial part of the macrophage in obesity, we found that *ApoE* mRNA expression with a trend higher in obese and diabetic human subjects (Fig. 1h). Notably, we found a substantial increase in plasma LPS levels after a 10-week HFD feeding (Fig. 1i). To verify further the reduction of *ApoE* in human obese subjects and mice models, we treated the peritoneal macrophages (PM) and bone marrow-derived macrophages (BMDM) with LPS (100 ng/mL) for 4 h. As shown in Fig. 1j and k, *ApoE* mRNA levels predominantly decreased after LPS stimulation in both primary macrophages. These results indicate that ApoE was negatively regulated by obesity and adipose tissue inflammation in humans and mice.

### ApoE deficiency prevents mice from being susceptible to HFD-induced obesity

**Fig. 2:**
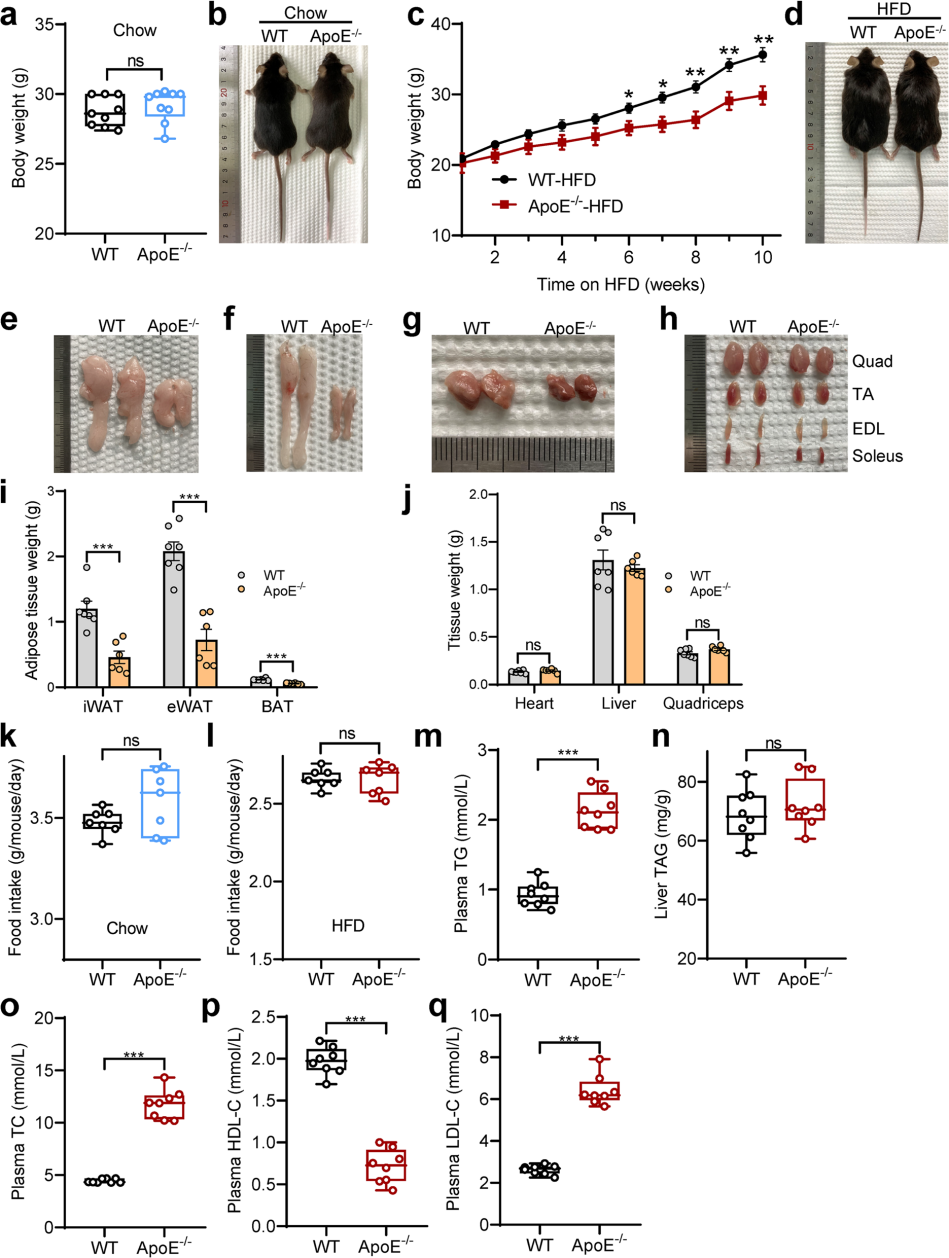
Loss of APOE modulates lipid metabolism during diet-induced obesity. **a** Body weight in chow-fed WT and ApoE^-/-^ mice (*n* = 9). **b** Representative images of WT and ApoE^-/-^ mice fed with chow for 12 weeks. **c**, **d** Body weight progression and representative pictures of WT and ApoE^-/-^ mice fed with HFD (*n* = 6–7). **e**-**h** Representative images of eWAT (**e**), iWAT (**f**), iBAT (**g**), and muscles (**h**) of WT and ApoE^-/-^ mice fed with HFD. **i**, **j** Tissue weight following HFD-fed for 10 weeks. **k**, **l** Averaged daily food intake of mice from chow (**k**) and HFD-fed (**l**) mice (*n* = 6–7). **m**, **n** Triglyceride in plasma and liver from HFD-fed WT and ApoE^-/-^ mice. **o**-**q** Plasma total cholesterol (TC), HDL cholesterol (HDL-C), and LDL cholesterol (LDL-C) in WT and ApoE^-/-^ mice fed with HFD (*n* = 8). All data represent mean ± SEM. *P < 0.05, **P < 0.01, ***P < 0.001, ns, no significance

To examine the in vivo impact of ApoE deficiency on obesity development, we subjected the mice to HFD feeding. To our surprise, we found that ApoE^-/-^ mice gained significantly less body weight than age-matched WT mice on HFD (Fig. 2c, d), but these differences disappeared on a standard chow diet for 12 weeks (Fig. 2a, b). Furthermore, ApoE^-/-^ mice exhibited lower epididymal WAT (eWAT), inguinal WAT (iWAT), and interscapular BAT (BAT) than WT following HFD feeding (Fig. 2e-g, i) but not heart liver, and muscles (Fig. 2h, j) such as quadriceps (Quad), tibialis anterior (TA), extensor digitorum longus (EDL), and soleus. These observations suggest that ApoE^-/-^ mice resist HFD-induced obesity. In line with this, no differences were observed in WT and ApoE^-/-^ mice’s daily average food consumption in chow (Fig. 2k) and HFD feeding (Fig. 2l). We next investigated whether the lower fat mass of ApoE^-/-^ mice in HFD results in lipid levels changes in plasma or liver. Levels of triglyceride (TG) in plasma were higher in ApoE^-/-^ mice (Fig. 2m), whereas liver TG levels remained similar between the groups (Fig. 2n). Measurements of crucial circulating lipid profiles indicated that plasma levels of total cholesterol (TC), HDL cholesterol, and LDL cholesterol content were markedly affected by ApoE abrogated (Fig. 2o-q). Together, these data demonstrated that inactivation of ApoE ameliorates HFD-induced obesity but facilitates lipid dys-homeostasis diseases such as atherosclerosis.

### ApoE deficiency protects mice from HFD-induced glucose intolerance and insulin resistance

**Fig. 3:**
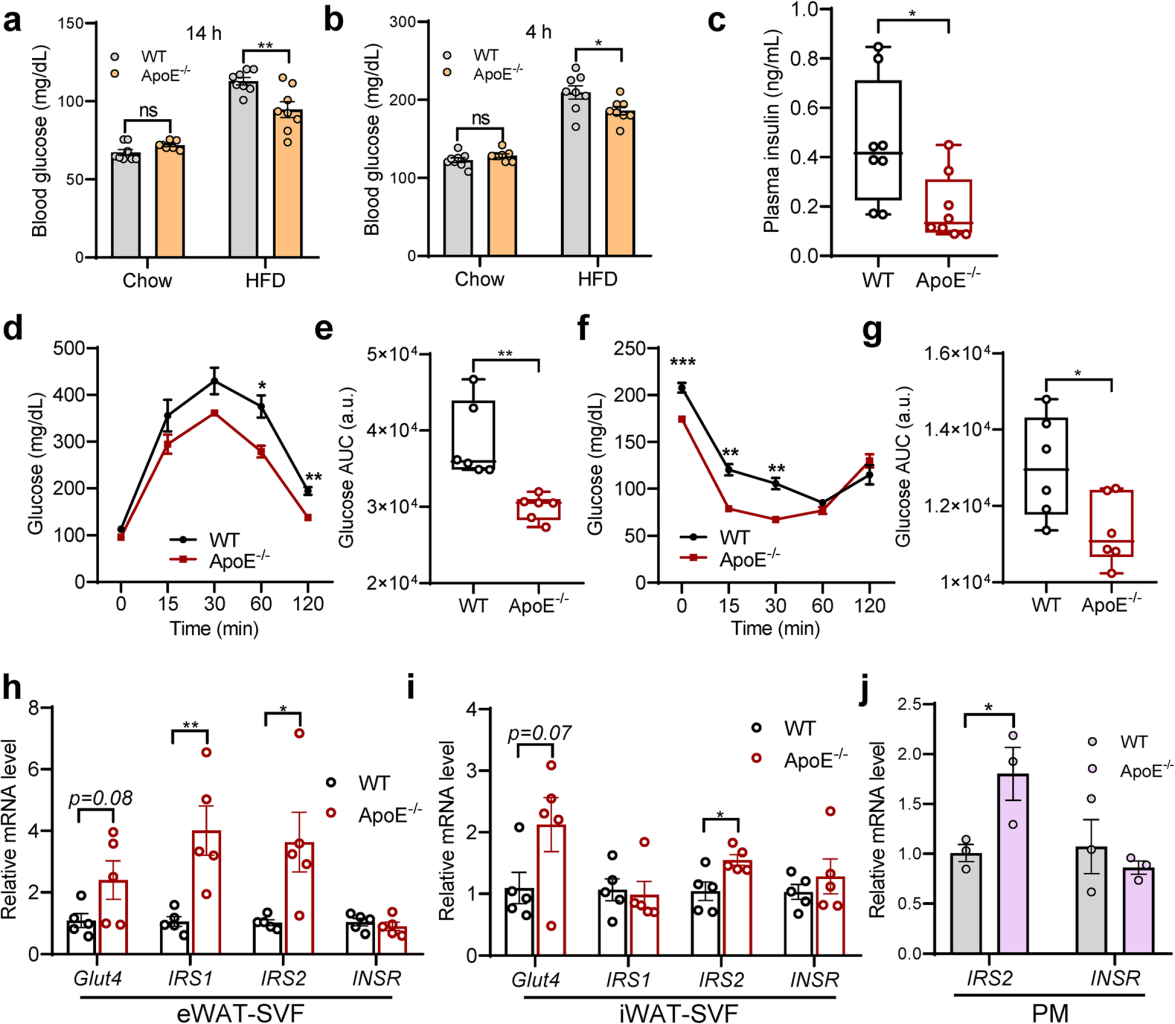
ApoE abrogation ameliorates HFD-induced glucose clearance. **a**, **b** Different during fasting blood glucose from chow and HFD-fed mice at the indicated time (*n* = 8). **c** Basal plasma insulin levels after overnight fasting (*n* = 8). **d**, **e** GTT (**d**) and AUC (**e**) in WT and ApoE^-/-^ mice fed with HFD for 12 weeks. **f**, **g** ITT (**f**) and AUC (**g**) in WT and ApoE^-/-^ mice fed with HFD for 12 weeks (*n* = 6). **h**, **i** Transcription analysis of Glut4, IRS1, IRS2, and INSR in SVF of eWAT (**h**) and iWAT (**i**) from WT and ApoE^-/-^ mice. **j** Transcription analysis of IRS2 and INSR in in LPS-stimulated PM (100 ng/mL, 24 h, *n* = 3). PM, peritoneal macrophages. Data represent mean ± SEM. *P < 0.05, **P < 0.01, ***P < 0.001, ns, no significance

ApoE is a multifunctional lipoprotein with central roles in lipid metabolism, and our studies found that ApoE may be a key regulator in diet-induced obesity and lipid homeostasis. As such, we next determine whether ApoE ablation might promote negative glucose balance in obesity. As shown in Fig. 3a and b, overnight or short fasting blood glucose levels were unexpectedly lower in ApoE^-/-^ HFD-fed mice, whereas similar results were absent in the chow diet. Moreover, HFD-induced hyperinsulinemia was also alleviated in ApoE^-/-^ mice (Fig. 3c). Glucose tolerance test (GTT) and insulin tolerance test (ITT) indicated that ApoE^-/-^ mice following HFD feeding exhibited significantly improved glucose tolerance (Fig. 3d and e) and insulin sensitivity (Fig. 3f and g). To more comprehensively investigate the role of ApoE in glucose homeostasis and insulin sensitivity in HFD-fed mice, we assessed the expression levels of critical genes involved in insulin signaling pathways and glucose transport, including Glut4, IRS1, IRS2, and insulin receptor (INSR). As illustrated in Fig. 3h-j, these genes were differentially induced in the adipose tissue stromal vascular fraction (SVF) of eWAT and iWAT and PM from ApoE^-/-^ mice while absent in INSR, possibly due to tyrosine kinase properties. Furthermore, we observed varying degrees of upregulation in the expression of these genes in adipocytes from ApoE^-/-^ mice (Supplementary Fig. 3a and b). Collectively, these findings define a predominant role of ApoE in orchestrating metabolic homeostasis partly through improved glucose tolerance and insulin sensitivity.

### ApoE deficiency exacerbates HFD-induced adipose tissue hypertrophy and inflammation in mice

**Fig. 4:**
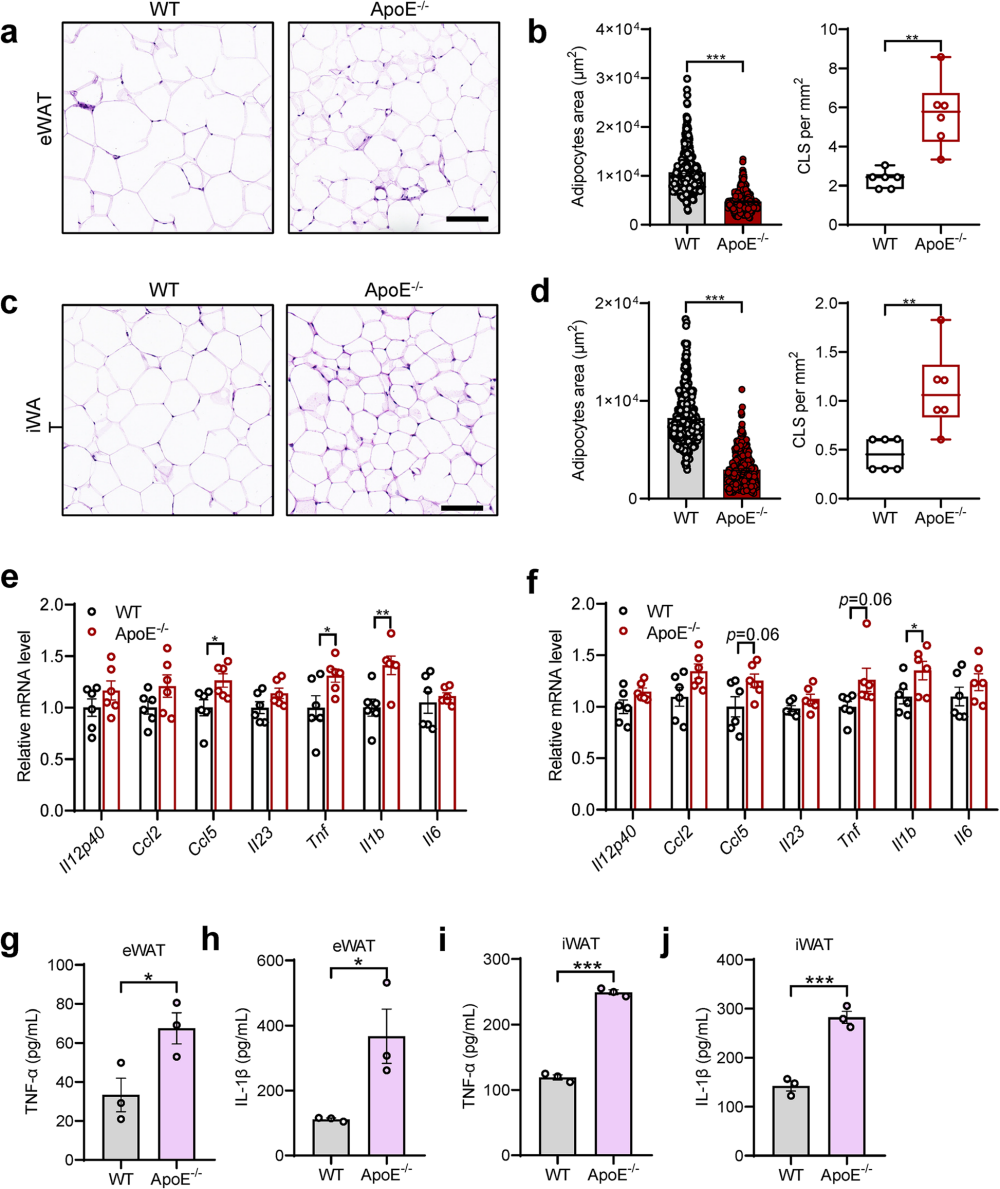
ApoE deficiency aggravates HFD-induced metabolic inflammation in adipose tissues. **a**, **b** Representative H&E-stained sections (**a**) and quantitative results of adipocytes area and CLS (**b**) of eWAT from WT and ApoE^-/-^ HFD feeding mice (*n* = 6). Scale bars, 100 μm. **c**, **d** Representative H&E staining images (**c**) and quantification of adipocytes area and CLS (**d**) of iWAT from WT and ApoE^-/-^ HFD feeding mice (*n* = 6). Scale bars, 100 μm. **e**, **f** QPCR analysis of proinflammatory genes in eWAT (**e**) and iWAT (**f**) from WT and ApoE^-/-^ mice on HFD (*n* = 6). **g**, **h** TNF-α and IL-1β levels in supernatants of eWAT cultures determined by ELISA (*n* = 3). **i**, **j** TNF-α and IL-1β levels in supernatants of iWAT cultures determined by ELISA (*n* = 3). Data represent mean ± SEM. *P < 0.05; **P < 0.01; ***P < 0.001

We found that ApoE deficiency protects mice from HFD-induced obesity; however, it is well-known that ApoE^-/-^ mice show a marked increase in TC and TG. To understand the nature of the adipose tissue physiology that ApoE influences, we performed hematoxylin and eosin (H&E) staining. ApoE^-/-^ mice displayed a more beneficial phenotype with smaller adipocytes but more CLS in eWAT and iWAT (Fig. 4a-d). To confirm the role of ApoE in inflamed adipose tissue, which promotes the IR of the system, inflammatory gene expression of adipose tissue was analyzed, and increased expression of *Tnf* and *Il1b* were observed both in eWAT (Fig. 4e) and iWAT (Fig. 4f) in the ApoE^-/-^ mice. In line with the above results, adipose tissues from ApoE^-/-^ mice showed higher TNF-α and IL-1β production than in WT on a HFD (Fig. 4g-j). These data prove that ApoE deficiency contributes to adipose tissue inflammation and dysfunction during obesity.

### Depletion of ApoE expedites the transcriptional initiation of proinflammatory gene expression within adipose tissue SVF and macrophages

**Fig. 5:**
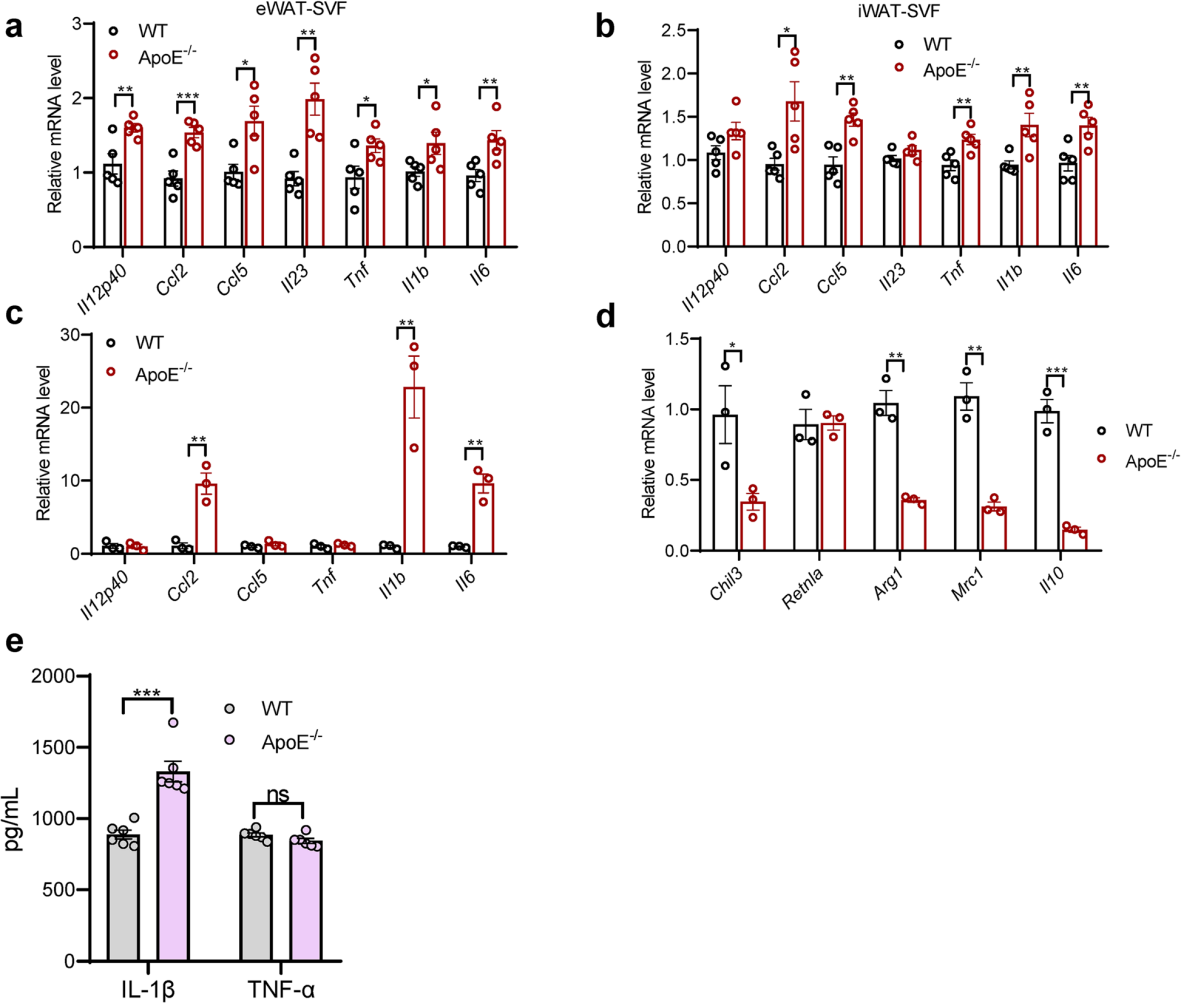
Depletion of ApoE expedites the transcriptional initiation of proinflammatory gene expression in SVF and macrophages. **a**, **b** mRNA levels of indicated proinflammatory genes in eWAT and iWAT SVF from indicated mice fed HFD (*n* = 5). **c** mRNA levels of classically activated macrophage marker in PM following exposure to LPS (100 ng/mL) for 24 h (*n* = 3). **d** mRNA levels of alternatively activated macrophage marker in PM following exposure to IL-4 (20 ng/mL) for 24 h (*n* = 3). **e** IL-1β and TNF-α levels in culture supernatants from LPS-primed (500 ng/mL) PM followed with ATP (2 mM) for 45 min (*n* = 3)

To further determine whether the immune cell population is the critical factor for adipose tissue inflammation and dysfunction, we isolated SVF to exclude the mature adipocytes for subsequent QPCR assays. Consistent with whole tissue results, QPCR analysis revealed a marked increase of proinflammatory cytokines in adipose tissue SVF from HFD-fed mice (Fig. 5a, b). Given that macrophages are the primary cell type contributing to the CLS formation and inflammation during the expansion of the adipose tissue, we assessed whether ApoE loss might directly impact macrophage polarization. In agreement with SVF profile findings, PM from ApoE^-/-^ mice had robust transcriptional induction of M1-like genes and significantly decreased M2-like genes (Fig. 5c, d). Meanwhile, ApoE deficiency in macrophages significantly increased the levels of IL-1β but not TNF-α (Fig. 5e) in the culture supernatant. This finding suggests that the observed alterations resulting from ApoE knockout may be associated with independent splicing and maturation of IL-1β.

###  NLRP3 deletion curbs macrophage activation of ApoE^-/-^ mice

**Fig. 6:**
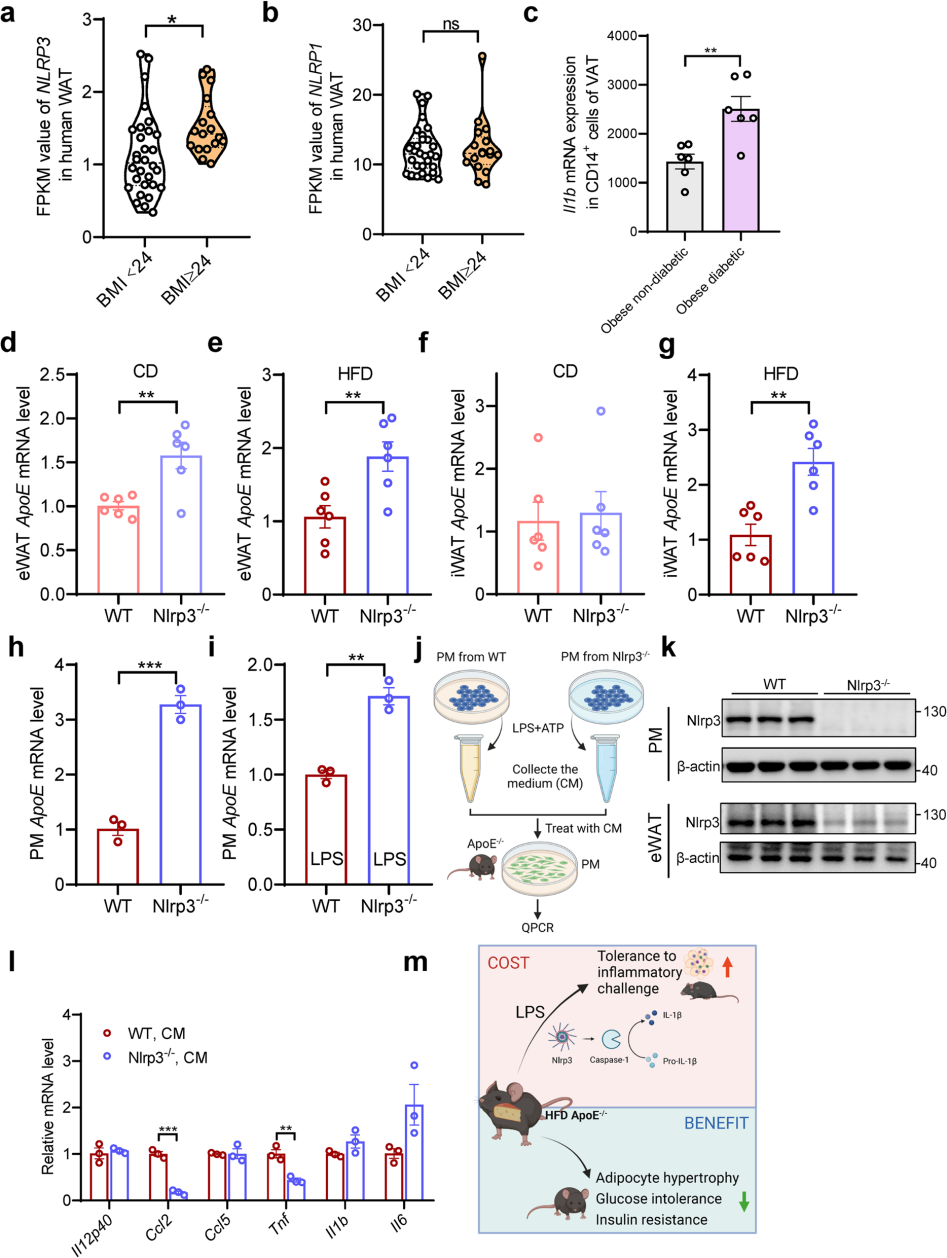
NLRP3 inflammasome is required for proinflammatory macrophage activation in mice. **a**, **b** The FPKM value of *NLRP3 and NLRP1* in indicated groups from RNA-Seq of human OAT (*n* = 30, BMI < 24; *n* = 17, BMI ≥ 24). **c** Previous human samples data of *Il1b* expression were collected from the GEO dataset (GSE54350, *n* = 6). **d**-**g** Transcription analysis of *ApoE* in Ewat and iWAT under chow diet (d, f) or HFD (e, g) from WT and Nlrp3^-/-^ mice (*n* = 6). **h**, **i** Transcription analysis of *ApoE* in (**i**) or absent (**h**) LPS-stimulated PM (100 ng/mL, 24 h, *n* = 3). **j** Schematic depicting PM treatment with LPS&ATP-stimulated conditioned medium harvested from PM of WT and Nlrp3^-/-^ mice. **k** NLRP3 expression in PM and eWAT from WT and Nlrp3^-/-^ mice were determined by Western blot (*n* = 3, each lane represents one independent biological sample). **l** QPCR analysis of inflammatory gene expression in PM of ApoE^-/-^ mice treated with CM from indicated groups. **m** Schematic model depicting the ApoE manipulates inflammation and metabolism. OAT, omental adipose tissue. CD, chow diet. HFD, high-fat diet. PM, peritoneal macrophages. Data represent mean ± SEM. *P < 0.05, **P < 0.01, ***P < 0.001, ns, no significance

Given the critical roles of NLRP3 and NLRP1 inflammasomes in the maturation of IL-1β, we further performed correlation analysis of NLRP3 and NLRP1 expression with BMI and found a significant increase of NLRP3 in overweight human subjects (Fig. 6a). At the same time, such induction was absent in NLRP1 (Fig. 6b). More specifically, the previous database of human samples from obese patients with diabetes showed that IL-1β was dramatically upregulated compared with obese non-diabetic subjects (Fig. 6c). These results indicate that the NLRP3-mediated IL-1β inflammation may be involved in macrophage activation in ApoE^-/-^ mice. To further investigate the relationship between ApoE and NLRP3, we examined the ApoE expression in eWAT and iWAT of Nlrp3^-/-^ mice under both standard chow diet (CD) and HFD. As depicted in Fig. 6d-g, Nlrp3^-/-^ mice displayed an upregulation of ApoE in eWAT under CD, and both eWAT and iWAT exhibited significant upregulation under HFD feeding. We further isolated peritoneal macrophages from WT and Nlrp3^-/-^ mice to confirm whether this change also happened in macrophages. The results show that, regardless of the presence of LPS, ApoE expression in PM from Nlrp3^-/-^ mice was significantly upregulated (Fig. 6h and i). These findings indicate that NLRP3 may exert negative feedback regulation on ApoE in chronic inflammatory microenvironments induced by overnutrition. Moreover, we utilized a conditioned medium of PM from WT and Nlrp3^-/-^ mice to study macrophage-related inflammation in vitro (Fig. 6j), and as shown in Fig. 6k and Fig. S4a-c, the expression of NLRP3 protein and mRNA levels were markedly reduced in both of PM and eWAT from Nlrp3^-/-^ mice. As expected, PM treatment with CM from NLRP3-deficient mice induced robust reductions in proinflammatory gene expression, such as *Ccl2* and *Tnf*, compared to CM from WT (Fig. 6i). These data demonstrate that NLRP3 but not NLRP1 is highly associated with ApoE deficiency induced macrophage activation.

## Discussion

Increasing evidence indicates that chronic low-grade metabolic inflammation dramatically contributes to the development of obesity-related metabolic disorders [19–21]. Nevertheless, the molecular mechanisms maintaining the immune cell’s function remain obscure. Here, we defined a crucial role for ApoE in protecting HFD-induced obesity, hyperglycemia, and IR but at an inflammatory cost in WAT, which provides new insights into the diagnostic and therapeutic approach for obesity. Notably, we demonstrated that ApoE acts as a regulator of inflammation through NLRP3 inflammasome. These findings revealed that ApoE is essential for obesity-related inflammation. The signature of *ApoE* involved in inflammation and lipid metabolism is found both in mice and humans across multiple obesity models, suggesting the pluripotent role of ApoE in the NLRP3-mediated inflammation induced by obesity.

ApoE is a multifunctional regulator protein for integrating signaling of neuron plasticity, cognition, and inflammation-related cardiovascular disease and fat metabolism [22–24]. In both obese human subjects and HFD-induced obese mice, as well as *db/db* mice, a marked downregulation of ApoE was observed in our work. Similarly, the PM had less ApoE expression under the LPS treatment. However, intriguingly, within the obese diabetic population (GSE54350), there was an inclination towards increased ApoE expression in CD14^+^ macrophage residing in the visceral adipose tissue (VAT) by microarray. We postulate that the elevated expression of ApoE in macrophages of individuals afflicted with obesity and diabetes may influence the regulation of chronic inflammation-associated glucose and lipid metabolism. Here, the elevation of plasma LPS levels in HFD-fed mice and the reduction in ApoE expression following LPS stimulation indicate that ApoE may regulate metabolism and inflammation induced by overnutrition. Notably, LPS from gut microbiota entering the circulation may be a possible mechanism of inflammation contributing to obesity [25]. A previous study characterized that ApoE^-/-^ mice are more resistant to becoming obese, and our work further highlights the complexity of ApoE in obesity-related chronic inflammation in adipose tissue [26]. Furthermore, we identified the NLRP3 but not NLRP1 inflammasome to be a significant driver of adipose tissue macrophage activation. Taken in aggregate, these findings propose a potential involvement of ApoE in the modulation of macrophages, consequently contributing to the pathogenesis of metabolic disorders associated with obesity.

We made the surprising discovery that ApoE^-/-^ mice gained lower body weight, WAT, and BAT weights compared to WT mice under HFD. Additionally, there was a notable improvement in HFD-induced glucose metabolism disorders and IR in ApoE^-/-^ mice. However, further investigations revealed that ApoE^-/-^ mice experienced aggravated adipose tissue inflammation, as evidenced by increased expression and secretion of inflammatory factors. Interestingly, despite elevated plasma lipid profiles in ApoE^-/-^ mice under HFD feeding, the hepatic triglyceride (TG) content did not change. These data indicate that the impact of ApoE deficiency on lipid metabolism is primarily associated with reverse cholesterol transport [27]. Here, we speculate that ApoE^-/-^ mice, at the cost of increased inflammation, partially maintain metabolic balance under HFD. Therefore, our findings provide insight into the mechanism macrophages regulate glucose and lipid metabolism in the context of ApoE ablation may be different.

Metabolic inflammation is closely associated with obesity and represents a critical factor in the etiology of metabolic disorders [28]. Notably, ATMs with multiple adaptive plasticity play a crucial role in orchestrating metabolic inflammation [29]. As the primary inflammatory cells, macrophages significantly influence obesity-induced dysregulation of glucose and lipid metabolism as well as IR. Restoring the disrupted polarization of macrophages emerges as a promising therapeutic avenue for targeted interventions in this context [4, 30]. Here, we demonstrated that the expression of proinflammatory genes *Ccl2*,* Ccl5*,* Il1b*, and* Il6* markedly increased only in the SVF rather than in adipocytes. Moreover, prominent crown-like structures (CLS) in adipose tissue HE staining suggests that the inflammatory response within the adipose tissue of ApoE^-/-^ mice is predominantly attributed to macrophage macrophages. In addition, *ApoE* mRNA expression is decreased in F4/80^+^CD11c^+^ ATMs, which have been reported to be sensitive to macrophage activation in obesity, which dynamically influences the tissue microenvironment [31]. Consistently, ApoE^-/-^ mice exhibited augmented increased gene expression in PM such as *Ccl2*,* Il1b*, and* Il6*, as well as the supernatant level of IL-1β, while TNF-α release remained unaffected, suggesting that ApoE deficiency induced the inflammasome activation stage but not the priming stage.

The cleavage of pro-IL-1β by inflammasomes is an essential requirement for IL-1β activation. To define the potential inflammasome implicated in the IL-1β production, RNA-Seq was performed to show the induced of NLRP3 but not NLRP1 in human obesity subjects. Also, more specifically, there was an increased expression of *Il1b* mRNA in CD14^+^ cells of VAT from microarray data [32]. NLRP3 is an innate immune sensor capable of instigating an inflammatory cascade in response to multiple stressors, such as overnutrition. Consequently, the regulation of NLRP3 inflammasome activation must be tightly controlled to ensure effective functioning while preventing excessive inflammatory responses [33]. Finally, we employed CM derived from PM of NLRP3^-/-^ mice to stimulate PM derived from ApoE^-/-^ mice. As expected, inflammatory gene expression no longer increased compared to CM from WT mice. Thus, we concluded that NLRP3-mediated IL-1β production might be partially responsible for the macrophage activation due to ApoE deficiency. We also aim to elucidate the specific process of inflammasome inhibition, such as assembly or activation. It is also necessary to clarify whether inhibition is mediated through receptors or ubiquitination of the inflammasome itself. However, it should be noted that we did not investigate adipose tissue T-cells, which have been previously implicated in obesity-related NLRP3 regulation [34].

Some limitations of this study should be noted. The specific mechanisms ApoE regulates inflammation via NLRP3 also require further investigation. Mice with conditional macrophage Nlrp3 knockout may be utilized to observe the inflammation levels related to ApoE deletion. Furthermore, we aim to increase sample sizes as much as possible in vitro to improve our study’s reliability. Our research did not explore the effects of ApoE overexpression in animals as we are uncertain whether macrophages are the sole cells through which ApoE exerts its influence. Future in vivo studies may investigate overexpression ApoE to confirm its anti-inflammatory effects in overnutrition.

In summary, our data demonstrated that ApoE ablation alleviates obesity and dysregulation of glucose metabolism at the expense of inciting chronic inflammation through NLRP3 inflammasome in obesity (Fig. 6m). We uncovered a previously unknown role of ApoE in obesity as a crucial mediator connecting inflammation and overnutrition, which advances the understanding of the instigation of chronic inflammation during obesity and broadens the arsenal for obesity and associated metabolic disorders.

## Materials and methods

### Human samples

Biopsies from visceral adipose tissue from human omental adipose tissue (OAT) were obtained from 47 individuals and with their informed written consent after the nature and possible consequences of the studies were explained. The studies on human OAT were approved by the Second Affiliated Hospital, Zhejiang University School of Medicine (Approval number: 2020-528). All participants undergo medical history inquiries before hospitalization and clinical chemistry analyses after an overnight fast. Human OAT was obtained from the patients who underwent surgery with an oblique inguinal hernia and gallstone at the Second Affiliated Hospital, Soochow University. Participants were between 20–84 years of age, with a BMI range of 17.8–34.69. All the samples were dissected and immediately frozen in liquid nitrogen for RNA extraction. The baseline characteristics of the participants are shown in Supplementary Tab. 1.

### Mice

Male C57BL/6J and APOE knockout (ApoE^-/-^) mice were generated and purchased from GemPharmatech Co. Ltd. (Jiangsu, China). Nlrp3 knockout (Nlrp3^-/-^, #021302, B6.129S6-Nlrp3tm1Bhk/J) mice were kindly provided by Pro. Zhexu Chi (Zhejiang University) and genotyped according to the standard protocols. The *db/db* (leptin receptor-deficient) mice were purchased from the Jackson Laboratory (#000697). ApoE^-/-^ mice primers genotyped: F: 5’TGCCTAGTCTCGGCTCTGAACTAC3’, R: 5’CAACCTGGGCTACACACTAATTGAG3’. All mice were maintained at a constant temperature of 23 ℃ and 50–70% humidity under 12 h light/12 h dark cycle. Mice fed with either standard normal rodent chow diet (CD, SLACOM, P1101F) or HFD (60% fat, D12492, Fanbo Biological Engineering Co., Ltd., Wuxi, China) for the indicated time. Mice were sacrificed, and tissues were removed and frozen in liquid nitrogen before further analysis. All animal studies were performed under a protocol approved by the University Committee on Use and Care of Animals at the Anhui Medical University (LLSC20221129) and followed the ARRIVE guidelines 2.0 [35].

### PM and BMDM primary cell culture

Mice peritoneal macrophages (PM) were collected 3 or 4 days after 4% thioglycolate (Millipore) injection. PM was cultured in DMEM (11995065, Gibco) supplemented with 10 % FBS (SE100-011, VisTech) and 1×streptomycin/penicillin (C0222, Beyotime) followed by stimulations. Mice bone marrow-derived macrophages (BMDM) were generated from the femurs and tibias of 6-week-old male mice as described [36]. BMDM grown in DMEM containing 10 % FBS and macrophage colony-stimulating factor (M-CSF) (15 ng/mL, 315-02-50, PeproTech). All cells were cultured in a humidified 5 % CO_2_ incubator at 37 ℃. Polarization of PM was induced for M1 activation with LPS (100 ng/mL) treatment or M2 activation with IL-4 (20 ng/mL) for 24 h before subsequent biochemical analysis. For LPS-induced PM medium collection, cells were plated and stimulated with LPS (500 ng/mL) for 4 h and ATP (2 mM) for 45 min subsequently, the conditioned medium (CM) was harvested for subsequent applications.

### Isolation of adipose tissue SVF

The adipose tissue SVF was collected as previously described [37, 38]. Briefly, mice were perfused with PBS through the left ventricle of the heart carefully to remove circulating leukocytes. Then, tissues were minced into small pieces and digested with HBSS containing 0.5% bovine serum albumin (BSA), collagenase D (11088815001, Roche), and Dispase II (D4693, Sigma) for about 40 min at 37 °C with constant agitation. After digestion, the tissue was filtered through a 70 μm cell strainer and spun down at 600 *g* for 10 min. SVF pellets and floating adipocytes were collected, and the SVF were resuspended in 1 mL RBC lysis buffer before further analysis.

### ELISA assays

Supernatants from adipose tissue culture were collected, and the concentrations of IL-1β (88-7013-88, Thermo) and TNF-α (88-7324-88, Thermo) were determined according to the manufacturer’s instructions.

### Histology

Adipose tissues (eWAT, iWAT, and BAT) were fixed in paraformaldehyde (4 %, PBS) for 24 h at 4 ℃, embedded with paraffin, and stained with H&E. H&E slides were analyzed with Pannoramic MIDI scanner (3D HISTECH). The crown-like structure (CLS) number of adipose tissues and adipocyte area were analyzed by CaseViewer software (3D HISTECH).

### Biochemical assays

Plasma LDL cholesterol (A113-1-1) and HDL cholesterol (A112-1-1) were quantified using a commercial determination kit (NanJing JianCheng Bioengineering Institute). Plasma triglyceride (TG) and total cholesterol (TC) content was measured with a biochemical analyzer. For liver TG analysis, 50 mg of liver tissues were homogenized (heptane isopropanol, 1:1 vol/vol), and supernatant were collected for determination with a triglyceride assay kit (BC0625, Solarbio). Mice plasma LPS was assessed with the Chromogenic Endotoxin Quant Kit according to the manufacturer’s instructions. (A39552, Thermo).

### Glucose tolerance and insulin tolerance tests

GTT and ITT were performed as previously described [39]. For the GTT, mice were intraperitoneally (IP) injected with D-(+)-glucose saline solution (1.6 g/kg body weight) after overnight fasting. Blood glucose levels were assessed before and at 15-, 30-, 60-, and 120-min following glucose injection via tail bleeds. For the ITT, Mice were fasted for 4 h and IP injected with insulin saline solution (1.2 Unit/kg body weight) for the ITT. Blood glucose levels were measured at the indicated time post insulin injection.

### Tissue culture

The mice isolated eWAT and iWAT tissues were washed in PBS, minced into fine pieces, and cultured in 6-well plates (0.5 g/well) in M199 medium supplemented with 5 % FBS, penicillin, and streptomycin [40]. After 24 h, culture supernatants were analyzed by ELISA for IL-1β and TNF-α.

### RNA preparation and quantitative PCR

Total RNA was extracted from homogenized tissues and cells using TRIzol (15596018, Invitrogen) reagent. For each sample, 1μg of RNA was reverse-transcribed using the Hifair III 1st Strand cDNA Synthesis SuperMix (11141ES60, Yeasen Biotech). Quantitative PCR assays were performed on a Bio-Rad CFX touch 96 using the Hieff qPCR SYBR Green Master Mix (11201ES08, Yeasen Biotech). Relative gene expression referred to the ∆∆Ct method and normalized to ribosomal protein P0 (Rplp0). Primer sequences are listed in Supplementary Tab. 2.

### Western blot

Tissues or whole cells were lysed and quantified. The primary antibodies used in this study were: β-actin (1:3000, AF7018, Affinity, RRID: AB_2839420), NLRP3 (1:1000, AG-20B-0014, AdipoGen, RRID: AB_2490202). The 2nd antibodies were: goat anti-rabbit IgG-HRP (1:8000, A6154, Sigma, RRID: AB_258284) and goat anti-mouse IgG-HRP (1:8000, A4416, Sigma, RRID: AB_258167). Protein levels were normalized to β-actin. Images were visualized using the Chemi-Doc MP system (Bio-Rad).

### Statistics

Statistical analyses were carried out using GraphPad Prism 8.0. Statistical differences were evaluated using a two-tailed unpaired Student *t* test for comparisons between two groups. For GTT and ITT, two-way ANOVA with multiple comparisons (Šídák) was used for statistical analysis. A *P* value ≤ 0.05 was considered statistically significant. No statistical method was used to predetermine the sample size. The experiments were not randomized and the investigators were not blinded to allocation during experiments as an assignment was based on genotypes.

### Supplementary Information

**Additional file 1: Fig. S1.** Representative tissue images and weight of WT and ApoE^-/-^ mice in chow diet. **Fig. S2.** QPCR analysis for verification of isolation SVF and mature adipocytes. **Fig. S3.** QPCR analysis of Glut4 and genes related to insulin pathway. **Fig. S4.** Generation of NLRP3 knockout (Nlrp3^-/-^) mice. **Supplementary Table 1.** The baseline characteristics of the participants. **Supplementary Table 2.** Primers used in this study. Raw gel data of Fig. 6k.

## Data Availability

The data of this study are available from the corresponding author upon reasonable request.
