# Supplementary material for: Apolipoprotein E (ApoE) orchestrates adipose tissue inflammation and metabolic disorders through NLRP3 inflammasome
Source: Mol Biomed. 2023 Dec 8;4:47. doi: 10.1186/s43556-023-00158-8 (PMC10703753; doi:10.1186/s43556-023-00158-8)

Apolipoprotein E (ApoE) orchestrates adipose tissue inflammation and metabolic disorders through NLRP3 inflammasome

Yulin Zhang[a,#], Ziwei Cheng[a,#], Liyu Hong[b,#], Jia Liu[a,#], Xinyue Ma[a], Wenjing Wang[a], Ran Pan[c], Wenjie Lu [a*], Qichao Luo[a*], Shan Gao[a*], Qin Kong[a*]

[a]*Department of Pharmacology, School of Basic Medical Sciences, Anhui Medical University, Hefei, 230032, China*

[b]*The Second Clinical Medical College, Anhui Medical University, Hefei, 230032, China*

[c]*RainbowFish Rehabilitation and Nursing School, Hangzhou Vocational and Technical College, Hangzhou, 310000, China*

***Corresponding authors:** kongqin@ahmu.edu.cn (Q. Kong), aydgs@126.com (S. Gao), luoqichao@ahmu.edu.cn (Q. Luo), wenjie63136@163.com (W. Lu)

Department of Pharmacology, School of Basic Medical Sciences, Anhui Medical University, Hefei, China.

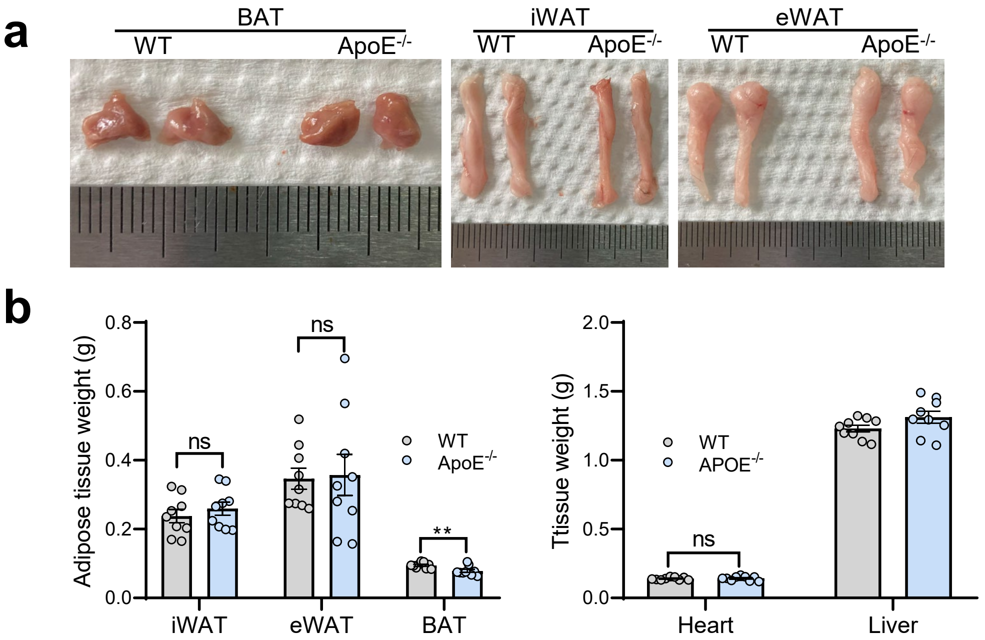

Fig. S1. Representative tissue images and weight of WT and ApoE$^{-/-}$ mice in chow diet. a Representative images of indicated tissues. **b** Tissue weight following chow-fed for 12 weeks (n=9). Data represent mean ± SEM. **$P < 0.01$, ns, no significance.

Figure S1

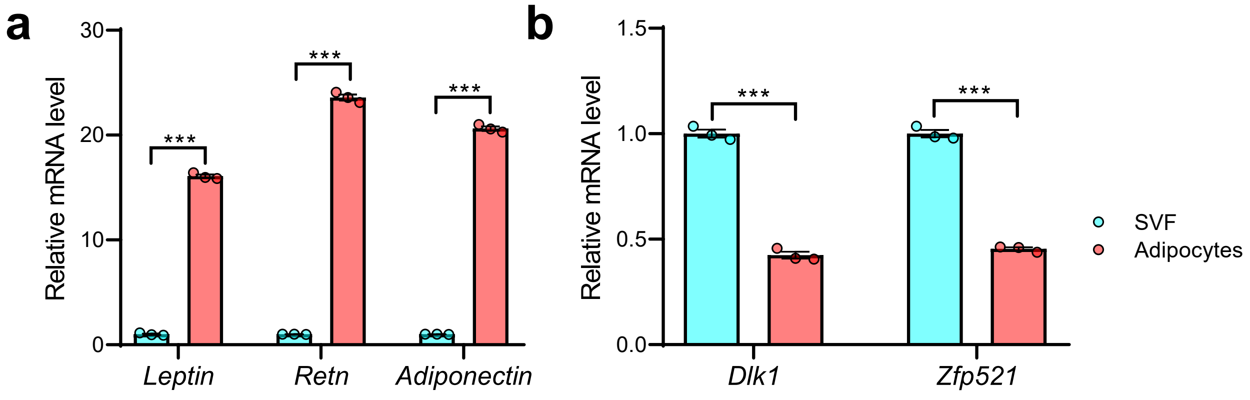

Fig. S2. QPCR analysis for verification of isolation SVF and mature adipocytes. a, b Representative marker gene expression of adipocytes (**a**) and SVF (**b**) isolated from WAT of WT mice (n=3). SVF, stromal vascular fraction. Data represent mean ± SEM. ***$P < 0.001$.

Figure S2

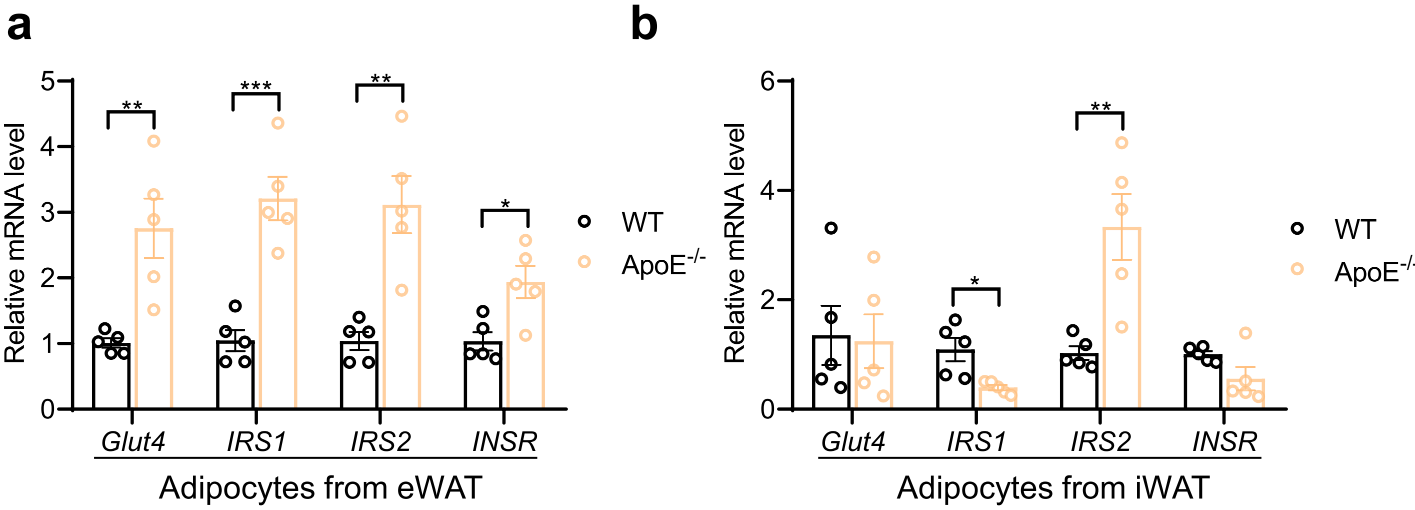

Fig. S3. QPCR analysis of Glut4 and genes related to insulin pathway. a, b Transcription analysis of Glut4, IRS1, IRS2, and INSR in adipocytes of eWAT (**a**) and iWAT (**b**) from WT and ApoE$^{-/-}$ mice. Data represent mean ± SEM. *$P < 0.05$, **$P < 0.01$, ***$P < 0.001$.

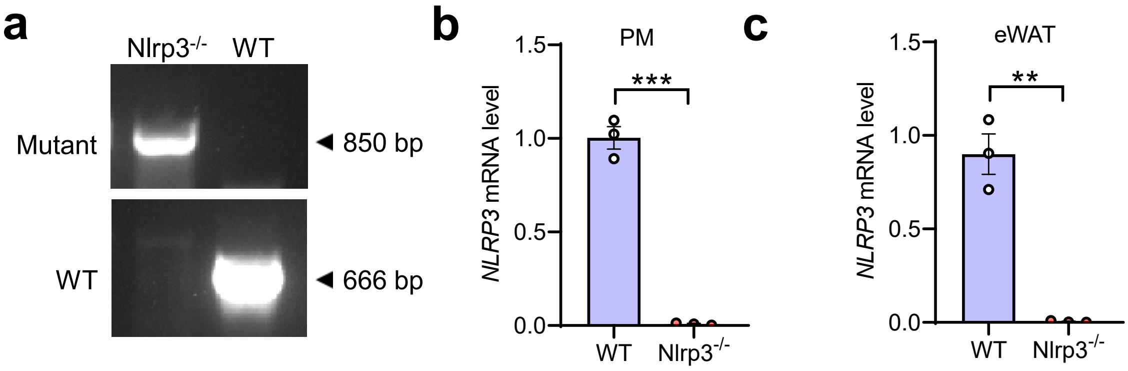

Fig. S4. Generation of NLRP3 knockout (Nlrp3$^{-/-}$**) mice. a** Genotypic PCR analysis of mice. **b, c** NLRP3 expression in PM (**b**) and eWAT (**c**) from WT and Nlrp3$^{-/-}$ mice were determined by QPCR (n=3). Data represent mean ± SEM. $^{**}P < 0.01$, $^{***}P < 0.001$.

Figure S3

Supplementary Table 1. The baseline characteristics of the participants

		(1) BMI < 24	(2) BMI≥24	*P*-value
Samples (n)		30	17	
Age		63.5. (13.8)	65.2 (9.59)	0.66
Sex, n (%)				
	Woman	6 (20%)	5 (29.4%)	-
	Man	24 (80%)	12 (70.6%)	-
BW (kg)		60.3 (10.6)	74.8 (14.6)	< 0.001
Height (cm)		164 (11.6)	167 (8.93)	0.46
BMI (kg/m^2)		21.3 (1.82)	27.4 (2.86)	< 0.001
BG (mmol/L)		4.62 (0.47)	5.64 (1.56)	0.0016

Information of the human subjects in indicated groups. BW, body weight; BMI, body mass index; BG, blood glucose. Data are represented as mean (SD) for age, BW, height, BMI and BG.

Supplementary Table 2. Primers used in this study

QPCR primers	5’→3’
Adiponectin-F	GCCCAGTCATGCCGAAGATGAC
Adiponectin-R	AGTGCCATCTCTGCCATCACGG
Apoe-F	CTGACAGGATGCCTAGCCG
Apoe-R	CGCAGGTAATCCCAGAAGC
Leptin-F	GAGACCCCTGTGTCGGTTC
Leptin-R	CTGCGTGTGTGAAATGTCATTG
Retn-F	ACAAGACTTCAACTCCCTGTTTC
Retn-R	TTTCTTCACGAATGTCCCACG
Zfp521-F	GGCTGTTCAAACACAAGCG
Zfp521-R	GCACATTTATATGGCTTGTTG
Dlk1-F	GCTGGGACGGGAAATTCTGCGA
Dlk1-R	AACCCAGGTGTGCAGGAGCA
Ccl2-F	AGGTCCCTGTCATGCTTCTG
Ccl2-R	TCTGGACCCATTCCTTCTTG
Ccl5-F	TGCCCACGTCAAGGAGTATTT
Ccl5-R	TTCTCTGGGTTGGCACACACT
Il6-F	AGTTGCCTTCTTGGGACTGA
Il6-R	TCCACGATTTCCCAGAGAAC
Il23-F	ATGCTGGATTGCAGAGCAGTA
Il23-R	ACGGGGCACATTATTTTTAGTCT
Tnf-F	AGCCCCCAGTCTGTATCCTT
Tnf-R	CTCCCTTTGCAGAACTCAGG
Il1b-F	TGGCAACTGTTCCTGAACTCAA
Il1b-R	AGCAGCCCTTCATCTTTTGG
Il12p40-F	CCAGAGACATGGAGTCATAG
Il12p40-R	AGATGTGAGTGGCTCAGAGT
Mrc1-F	CTCTGTTCAGCTATTGGACGC
Mrc1-R	CGGAATTTCTGGGATTCAGCTTC
Il10-F	GCCAAGCCTTATCGGAAATG
Il10-R	CACCCAGGGAATTCAAATGC
Chil3-F	GGGCATACCTTTATCCTGAG

Chil3-R	CCACTGAAGTCATCCATGTC
Arg1-F	ACACGGCAGTGGCTTTAACC
Arg1-R	TGGCGCATTCACAGTCACTT
Retnla-F	TACTTGCAACTGCCTGTGCTTACT
Retnla-R	TATCAAAGCTGGGTTCTCCACCTC
NLRP3-F	ATTACCCGCCCGAGAAAGG
NLRP3-R	TCGCAGCAAAGATCCACACAG
Rplp0-F	GAAACTGCTGCCTCACATCCG
Rplp0-R	GCTGGCACAGTGACCTCACACG
IRS1-F	TGGACATCACAGCAGAATGAAG
IRS1-R	AGACGTGAGGTCCTGGTTGT
IRS2-F	CACAACCTATCGTGGCACCT
IRS2-R	AAGGTCTCTGAACTGTGGCG
Glut4-F	GCTCTGACGTAAGGATGGGGA
Glut4-R	TTGTGGGATGGAATCCGGTC
INSR-F	TGTCCCCAGAAAAACCTCTTCA
INSR-R	AAGGGATCTTCGCTTTCGGG

Raw gel data of Figure 6k

Peritoneal macrophage, Nlrp3

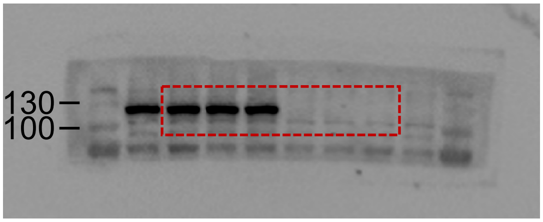

Peritoneal macrophage, β-actin

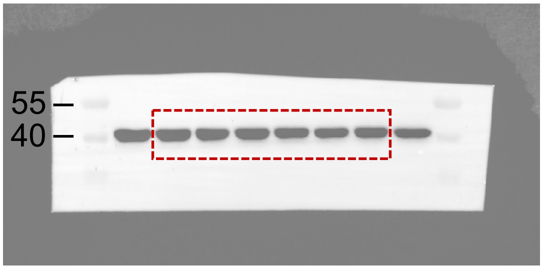

eWAT-Nlrp3

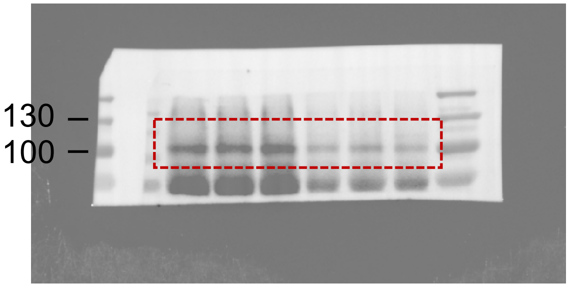

eWAT-β-actin

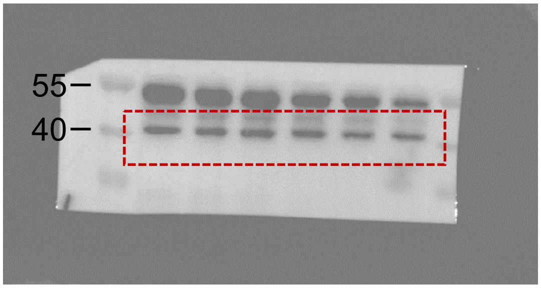

Supplement: Supplementary file 1 — Additional file 1: Fig. S1. Representative tissue images and weight of WT and ApoE-/- mice in chow diet. Fig. S2. QPCR analysis for verification of isolation SVF and mature adipocytes. Fig. S3. QPCR analysis of Glut4 and genes related to insulin pathway. Fig. S4. Generation of NLRP3 knockout (Nlrp3-/-) mice. Supplementary Table 1. The baseline characteristics of the participants. Supplementary Table 2. Primers used in this study. Raw gel data of Fig. 6k. [file 43556_2023_158_MOESM1_ESM.pdf]
